# Monitoring maternal and newborn health outcomes globally: a brief history of key events and initiatives

**DOI:** 10.1111/tmi.13313

**Published:** 2017-06-19

**Authors:** Ann-Beth Moller, Jane H. Patten, Claudia Hanson, Alison Morgan, Lale Say, Theresa Diaz, Allisyn C. Moran

**Affiliations:** 1 Department of Reproductive Health and Research (RHR) and UNDP-UNFPA-UNICEF-WHO-World Bank Special Programme of Research, Development and Research Training in Human Reproduction (HRP), World Health Organization, Geneva, Switzerland; 2 Green Ink, Oxford, UK; 3 Global Health, Department of Public Health Sciences, Karolinska Institutet, Stockholm, Sweden; 4 Maternal Sexual and Reproductive Health Unit, Nossal Institute for Global Health, University of Melbourne, Melbourne, Australia; 5 Department of Maternal, Newborn, Child and Adolescent Health, World Health Organization, Geneva, Switzerland

**Keywords:** maternal mortality, maternal morbidity, neonatal mortality, neonatal morbidity, stillbirths, monitoring, measurement, indicators, global initiatives, MMR, NMR, SBR

## Abstract

**Objective:**

Over time, we have seen a major evolution of measurement initiatives, indicators and methods, such that today a wide range of maternal and perinatal indicators are monitored and new indicators are under development. Monitoring global progress in maternal and newborn health outcomes and development has been dominated in recent decades by efforts to set, measure and achieve global goals and targets: the Millennium Development Goals followed by the Sustainable Development Goals. This paper aims to review, reflect and learn on accelerated progress towards global goals and events, including universal health coverage, and better tracking of maternal and newborn health outcomes.

**Methods:**

We searched for literature of key events and global initiatives over recent decades related to maternal and newborn health. The searches were conducted using PubMed/MEDLINE and the World Health Organization Global Index Medicus.

**Results:**

This paper describes global key events and initiatives over recent decades showing how maternal and neonatal mortality and morbidity, and stillbirths, have been viewed, when they have achieved higher priority on the global agenda, and how they have been measured, monitored and reported. Despite substantial improvements, the enormous maternal and newborn health disparities that persist within and between countries indicate the urgent need to renew the focus on reducing inequities.

**Conclusion:**

The review has featured the long story of the progress in monitoring improving maternal and newborn health outcomes, but has also underlined current gaps and significant inequities. The many global initiatives described in this paper have highlighted the magnitude of the problems and have built the political momentum over the years for effectively addressing maternal and newborn health and well-being, with particular focus on improved measurement and monitoring.

## Introduction

Every day, approximately 810 women die from preventable causes related to pregnancy and childbirth [1], almost 7000 newborns die [2] and more than 7000 babies are stillborn [3], based on the latest annual estimates. The vast majority of these deaths occur in lowand middle-income countries (LMICs). The burden of maternal and newborn morbidity remains more difficult to quantify (see definitions in Box 1). Over time, we have seen a major evolution of measurement initiatives, indicators and methods, such that monitoring efforts today use a wide range of maternal and perinatal indicators, and new indicators are under development, including indicators for morbidity (see Box 2).

Box 1 Definitions of terms

**Maternal death**: Death of a woman while pregnant or within 42 days of termination of pregnancy, irrespective of the duration and the site of the pregnancy, from any cause related to or aggravated by the pregnancy or its management, but not from accidental or incidental causes [4].

**Maternal morbidity**: Any health condition attributed to and/or complicating pregnancy and childbirth that has a negative impact on the woman’s well-being and/or functioning [5].

**Maternal near miss (MNM)**: A woman who nearly died but survived a complication that occurred during pregnancy, childbirth or within 42 days of termination of pregnancy [6].

**Neonatal death**: A death that occurs during the neonatal period – the first 28 days of life.

- **Late neonatal death**: A death that occurs in the late neonatal period (days 8–28).
- **Early neonatal death**: A death that occurs in the early neonatal period (days 1–7) [7].

**Neonatal morbidity**: No standard definition exists; there is ongoing work in this area.

**Perinatal death**: A death that occurs in the antepartum (before the onset of labour), intrapartum (during labour but before delivery) or early neonatal period (days 1–7); the definition may also be extended to refer to deaths throughout the neonatal period (days 1–28) [7,8].

**Pre-term birth:** Babies born alive before 37 weeks of pregnancy are completed.

- **Moderate to late pre-term**:32 to < 37 weeks.
- **Very pre-term**: 28 to < 32 weeks.
- **Extremely pre-term**: < 28 weeks [9].

**Pregnancy-related death, also known as a death occurring during pregnancy, childbirth and puerperium**: The death of a woman while pregnant or within 42 days of termination of pregnancy, irrespective of the cause of death (obstetric and non-obstetric) [10].

**Reproductive health**: A state of complete physical, mental and social well-being and not merely the absence of disease or infirmity, in all matters relating to the reproductive system and to its functions and processes [11].

**Stillbirth or foetal death**: A foetal death or stillbirth is defined as a baby born with no signs of life after a specified threshold [12]. For international comparison, WHO defines a stillbirth according to the 10th edition of the International Classification of Diseases (ICD-10) definition of late foetal death (see below).

- **Intrapartum (or fresh) foetal death (or stillbirth)**: Occurring after the onset of labour and before birth [7].
- **Antepartum (or macerated) foetal death (or stillbirth)**: Occurring before the onset of labour [7].
- **Late foetal death**: A foetal death weighing at least 1000 g, or (if the birthweight is not available) a gestational age of 28 completed weeks or more, or a crown–heel length of 35 cm or more (ICD-10 definition) [7,12].
- **Early foetal death**: A foetal death weighing at least 500 g, or (if birthweight is not available) a gestational age of 22 completed weeks or more, or a crown–heel length of 25 cm or more (ICD-10 definition) [7,12].

Box 2 Relevant indicators*

A **MORTALITY**

***Maternal:***

- **Maternal mortality ratio (MMR)** – Number of maternal deaths during a given time period per 100,000 live births during the same time period [1].
- **Institutional maternal mortality ratio** – Number of maternal deaths among 100,000 deliveries in health facilities/institutions [13].
- **Maternal mortality rate (MMRate)** – The MMRate ‘is found by dividing the average annual number of maternal deaths in a population by the average number of women of reproductive age (typically those aged 15 to 49 years) who are alive during the observation period. Thus, the MMRate reflects not only the risk of maternal death per pregnancy or per birth, but also the level of fertility in a population’ [14].
- **Adult lifetime risk of maternal death** – The probability that a 15-year-old girl will die eventually from a maternal cause [1,14].
- **The proportion of deaths among women of reproductive age that are due to maternal causes (PM)** – The number of maternal deaths in a given time period divided by the total deaths among women aged 15–49 years [1].

***Neonatal/child:***

- **Neonatal mortality rate (NMR)** – Probability that a child born in a specific year or period will die in the first 28 days of life (0–27 days) if subject to age-specific mortality rates of that period, expressed per 1000 live births [13].
- **Under-five mortality rate (U5MR)** – The probability of a child born in a specific year or period dying before reaching the age of 5 years, if subject to age-specific mortality rates of that period, expressed per 1000 live births [13]. (Neonatal deaths make up a portion of this.)
- **Infant mortality rate (IMR)** – The probability that a child born in a specific year or period will die before reaching the age of 1 year, if subject to age-specific mortality rates of that period, expressed as a rate per 1000 live births [13]. (Neonatal deaths make up a portion of this.)

***Stillbirths:***

- **Stillbirth rate (SBR)** – Number of stillbirths per 1000 total births. Stillbirths can occur antepartum or intrapartum. In many cases, stillbirths reflect inadequacies in antenatal care coverage or in intrapartum care. For purposes of international comparison, stillbirths are defined as third trimester foetal deaths (≥ 1000 g or ≥ 28 weeks) [13].

B **MORBIDITY**

***Maternal:***

- **Maternal morbidity rate or ratio** – No indicator exists; there is ongoing research in this area.
- **Maternal near miss (MNM) incidence ratio** – Number of MNM cases per 1000 live births [6].
- **Severe systemic infection/sepsis in the post-natal period (%)** – Percentage of women in health facilities with severe systemic infection/sepsis in the post-natal period, including readmissions (after birth in a facility) [13].

***Neonatal:***

- **Neonatal morbidity rate or ratio** – No indicator exists; there is ongoing research in this area.
- **Incidence of low birthweight among newborns** – Percentage of live births that weigh less than 2500 g [13].
- **Pre-term birth rate** – All live births before 37 completed weeks (whether singleton or multiple) per 100 live births [9].
- **Newborns receiving essential newborn care** – Percentage of newborns who received all four elements of essential newborn care: immediate and thorough drying, immediate skin-to-skin contact, delayed cord clamping and initiation of breastfeeding in the first hour [13].
- **Treatment for neonatal sepsis** – Newborns with suspected severe bacterial infection who receive appropriate antibiotic therapy [13].

C **OTHER (Relevant to both mortality and morbidity)**

***Maternal:***

- **Antenatal care coverage** – Percentage of women aged 15 –49 years with a live birth in a given time period who received antenatal care, four times or more times from any provider [13,15].
- **Post-partum care coverage – women** – Percentage of women who have post-partum contact with a health provider within two days of delivery [13,15].
- **Maternal death review coverage (%)** – Percentage of maternal deaths occurring in the facility that were audited and reviewed [13].
- **Proportion of women in antenatal care who were screened for syphilis during pregnancy** [15]. ***Neonatal:***
- **Post-natal care coverage – newborn** – Proportion of newborns who have a post-natal contact with a health provider within two days of delivery [13,15].
- **Neonatal death review coverage (%)** – Percentage of neonatal deaths occurring in the facility that were audited [13].
- **Proportion of infants who were breastfed within the first hour of birth** [15]. ***Childbirth/delivery:***
- **Births attended by skilled health personnel** – Percentage of live births attended by skilled health personnel during a specified time period [13].
- **Institutional delivery coverage** – Proportion of women who gave birth in a health institution (number of deliveries in institutions among total deliveries) [13].
- **Caesarean section rate** – Percentage of deliveries by caesarean section [13].

*For further analysis of maternal and newborn indicators, please see Moller *et al*., 2018 [16].

The growth of the world’s population, from under 2 billion 100 years ago to approximately 7.5 billion now [17], clearly reflects significant improvements in global health – especially child survival – raising life expectancies. But when the rate of world population growth peaked in the 1960s, it began to cause alarm, prompting re-assessments of global development and intensified efforts to monitor international population dynamics. This brought to light gaping disparities in mortality rates between regions and populations.

By this time, maternal mortality in higher-income countries had been measured over many decades, with high rates beginning to fall in the 1930s, converging at a maternal mortality ratio (MMR) of around 60 per 100,000 live births in 1960 [18]. But MMR gains in lower-income countries have lagged far behind [1], despite progress in health care and a halving of the global total fertility rate from 5 in 1950–1960 to approximately 2.5 currently [19]. By 2000, when the MMR was estimated at 16 versus 378, in more versus less developed regions [1], this was recognised as the largest disparity in any mortality between higher- and lower-income countries [18].

Monitoring of global progress in health and development has been dominated since before the turn of the millennium by efforts to set, measure and achieve global goals and targets: first the Millennium Development Goals (MDGs; 1990–2015) and subsequently the Sustainable Development Goals (SDGs; 2016–2030). Maternal health and child health were the focus of two of the eight MDGs (MDGs 4 and 5), and they are currently addressed under SDG3 on health and well-being.

Table 1 presents the relevant MDG and SDG targets and summarises MDG-era progress. By 2015, the 44% decline in global MMR had missed the ambitious target of 75%, and MMR was still almost 20 times higher in low- versus high-income countries [20]. The SDG target to reduce the global MMR below 70 by 2030 presents a serious challenge. Based on the latest available evidence from a 2014 WHO systematic analysis of global data on causes of maternal death for 2003 to 2009, 72.5% of 2,443,000 maternal deaths were due to direct obstetric causes (haemorrhage 27.1%, hypertension 14.0%, sepsis 10.7%, abortion 7.9%, embolism 3.2%, other 9.6%), and 27.5% were due to indirect causes (HIV-related 5.5%, pre-existing medical condition 14.8%, other 7.2%) [21]. Meanwhile, the under-five mortality rate (U5MR) was reduced by 55% worldwide, falling short of the MDG target to reduce it by two-thirds [2]. The global neonatal mortality rate (NMR) fell by 48% in the same period, to 19.1 per 1000 live births, although there was no MDG target [2]. Focusing on newborns will be critical to further reductions in child mortality, given that in 2015 almost half (47%) of under-five deaths were neonatal deaths [2]. The SDG target is an NMR in all countries of 12 or fewer. Based on global data for 2017, neonatal deaths are caused mainly by complications of prematurity (34%), intrapartum conditions (24%) and infections (20%), notably sepsis and acute respiratory infection [22,23]. Stillbirths have not been addressed by the MDGs or the SDGs, but the stillbirth rate (SBR) declined by 25.5% between 2000 and 2015 to 18.4 stillbirths per 1000 total births [12]. A target stated in the 2014 Every Newborn Action Plan is for all countries to reach an SBR of 12 or fewer by 2030 [24]. Most stillbirths are caused by ‘preventable conditions such as maternal infections (notably syphilis and malaria), non-communicable diseases and obstetric complications. Few are due to congenital disorders, but some of these are also preventable’ [25].

**Table 1:** Global goals and summary data

MDG targets for 2015 MDG 5 – Improve maternal health; MDG 4 – Reduce child mortality	Percentage gains per target during the MDG era (from 1990 to 2015) 2015 data in bold	SDG targets for 2030 SDG 3 – Ensure healthy lives and promote well-being for all at all ages
Maternal mortality		
MDG Target 5.A: Reduce by three quarters, between 1990 and 2015, the maternal mortality ratio (MMR)	5.1 MMR reduced by* World: 44% (from 385 to 216 deaths per 100,000 live births) Developing regions: 44% (from 430 to 239) Developed regions: 48% (from 23 to 12) 5.2 Proportion *increased by*†	SDG Target 3.1: By 2030, reduce the global MMR to less than 70 per 100,000 live births
*Indicators:*	World: 28% (from 59% to 75.4%)	*Indicators:*
*5.1 MMR per 100,000 live births;*	Developing regions: 28% (from 57% to 73.1%)	3.1.1 *MMR;*
*5.2 Proportion of births attended by skilled health personnel (%)*	Developed regions: 1990 data not found. 98.7% in 2015	*3.1.2 Proportion of births attended by skilled health personnel*
Child mortality		
MDG Target 4.A: Reduce by two-thirds, between 1990 and 2015, the under-five mortality rate (U5MR) *Indicators:*	4.1 U5MR reduced by‡ World: 53.3% (from 91 to 43 per 1000 live births) Developing regions: 54% (from 100 to 47) Developed regions: 60% (from 15 to 6) 4.2 IMR reduced by‡	SDG Target 3.2.1:§ By 2030, end preventable deaths of children under 5 years of age, with all countries aiming to reduce under-5 mortality to at least as low as 25 per 1000 live births
*4.1 U5MR per 1000 live births;*	World: 49.2% (from 63 to 32 per 1000 live births)	
*4.2 Infant mortality rate (IMR) per 1000 live births*	Developing regions: 49.3% (from 69 to 35) Developed regions: 58.3% (from 12 to 5)	*Indicator:* *3.2.1 U5MR*
Neonatal mortality		
No MDG target or other global target was set for neonatal mortality for 2015 *Indicator*:	4.1 U5MR reduced by‡ World: 47% (from 36 to 19 per 1000 live births) Developing regions: 48% (from 40 to 21)	SDG Target 3.2.2:§ By 2030, end preventable deaths of newborns, with all countries aiming to reduce neonatal mortality to at least as low as 12 per 1000 live births
*Neonatal mortality rate (NMR) per 1000 live births*	Developed regions: 63% (from 8 to 3)	*Indicator:* 3.2.2 *NMR* See also Box 4
Stillbirths		
No MDG target or other global target was set for stillbirths for 2015	SBR reduced from 2000k to 2015 by¶ World: 25.5% (from 24.7 to 18.4)	No SDG target was set for stillbirths, but targets were set by the Every Newborn Action Plan (ENAP; see Box 4]
*Indicator:*	Developed regions: 24.4% (from 4.5 to 3.4)	*Indicator:* SBR
*Stillbirth rate (SBR) per 1000 total births*	Sub-Saharan Africa (highest of all regions): 19.4% (from 35.6 to 28.7)	

As explained in the MDG report ‘Since there is no established convention for the designation of “developed” and “developing” countries or areas in the United Nations system, this distinction is made for the purposes of statistical analysis only’.

* Data from *Trends in maternal mortality*, WHO 2015 [20].

† 1990 data from *The Millennium Development Goals report 2015* [26]; 2015 data from *Progress towards the Sustainable Development Goals: Report of the Secretary-General*, Statistical annex: global and regional data for Sustainable Development Goal indicators (https://unstats.un.org/sdgs/files/report/2016/secretary-general-sdg-report-2016–Statistical-Annex.pdf).

‡ Data from *Levels and trends in child mortality: report 2015*, UNIGME 2015 [27].

§ Target 3.2 has been split in two (3.2.1 and 3.2.2) to separate out the targets for children (under 5 years old) and newborns (as was also done in the *Indicator and monitoring framework for the GSWCAH*).

¶ Data from Blencowe *et al*. 2016 [12] by MDG region.

**Figure 1:**
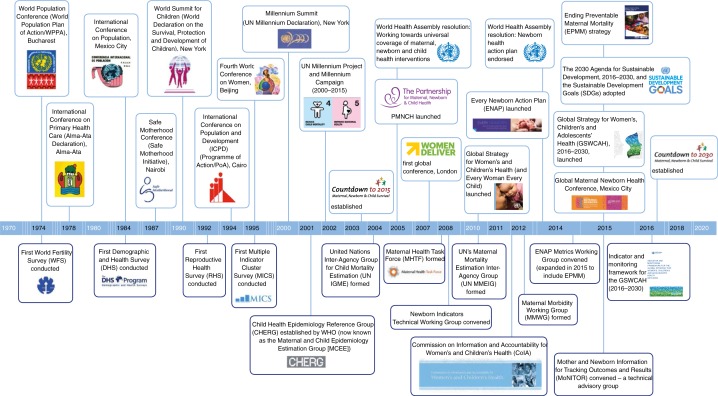
Key global initiatives and events related to maternal and newborn health.

The aim of this review was to support reflection and learning for accelerated progress and better tracking of maternal and newborn health outcomes, as well as progress towards the SDGs and universal health coverage. This review takes a global perspective, describing key global events and initiatives over recent decades to show how maternal and neonatal mortality – and, more recently, morbidity, as well as stillbirths – have been viewed, when they have achieved higher priority on the global health and development agenda, and how they have been measured, monitored and reported. The timeline presented in Figure 1 summarises these global initiatives. In addition, this review includes recommendations for accelerated action to achieve global goals and targets.

## Measuring, monitoring and prioritising maternal and perinatal mortality

### Establishment of the United Nations: global concern for maternal and child health

International recognition of maternal mortality as a critical issue began with the League of Nations, the forerunner to the UN, which was created in 1919 [28]. As reported by AbouZahr [29], ‘The League of Nations Health Section noted concerns about maternal mortality in 1930, reflecting the increasing interest in the topic in industrialised countries and the desire of many colonial powers to transfer to their colonies the benefits of medical progress that were by now so apparent’. The UN was established in 1945, and the League of Nations Health Organization became the WorldHealthOrganization(WHO). WHO’s Constitution entered into force in 1948, with one of its functions being ‘to promote maternal and child health and welfare’ [30].

### World conferences on population: early awareness, good intentions

Reflecting international concern about population growth since the 1960s, the UN has led three world conferences on population: in 1974 in Bucharest, Romania; in 1984 in Mexico City, Mexico; and in 1994 in Cairo, Egypt. While population, development and fertility were the focus of the World Population Plan of Action (WPPA – prepared by the UN Population Division [UNPD] before the first conference), one of the stated goals was to reduce mortality levels, particularly infant and maternal mortality levels, to the maximum extent possible in all regions of the world and to reduce national and sub-national differentials therein’; reduction of related maternal morbidity was also mentioned as requiring vigorous efforts. The WPPA stated clear international targets, including ‘countries with the highest mortality levels should aim by 1985 to have an expectation of life at birth of at least 50 years and an infant mortality rate of less than 120 per thousand live births’ [31]. These and other statements highlighted – in the early 1970s – the need to improve maternal and child health (MCH), to reduce mortality and morbidity, and to reduce disparities between higher- and lower-income regions. At the 1984 conference, by which time 123 countries were promoting family planning (up from 59 a decade earlier), recommendations were adopted for the further implementation of the WPPA [32], with more detailed information and more specific recommendations. But it was not until the 1994 conference (see ‘Key conferences’ below) that reproductive health and rights were truly put front and centre.

### The Alma-Ata Declaration and ‘Health for All’: sights set on the year 2000

Over forty years ago, in 1978, WHO and UNICEF convened the **International Conference on Primary Health Care**, which culminated with the **Alma-Ata Declaration**. A major 20th-century milestone in the field of public health, the Declaration, identified primary health care as the key to ‘Health for All’. This goal had been articulated in 1977 at the World Health Assembly (WHA): ‘the main social target of governments and of WHO should be the attainment of all the people of the world by the year 2000 of a level of health that will permit them to lead a socially and economically productive life’ [33]. Crucially, the Declaration identified MCH care, including family planning, as components of basic and essential primary health care [34]. In 1979, the WHA endorsed the Alma-Ata Declaration and launched the Global Strategy for Health for All by the Year 2000, which was formally adopted in 1981 [33].

### The Safe Motherhood Conference: the big push to reduce maternal mortality

While the importance of MCH had received verbal support at the UN population conferences and in the Alma-Ata Declaration, only child health interventions and family planning subsequently received major funding, from the United Nations Children’s Fund (UNICEF) and the United Nations Population Fund (UNFPA), respectively. This was part of the ‘selective primary healthcare’ strategy aimed at high coverage of interventions for the most important diseases in less developed regions, viewed as the most cost-effective approach to public health until ‘comprehensive primary health care’ could be available to all [35]. This led Rosenfield and Maine to publish their seminal 1985 paper declaring maternal mortality a neglected tragedy and asking ‘Where is the M in MCH?’ [36], cited the estimated 500,000 annual maternal deaths mentioned by the Director General of WHO during the WHA in 1979. The authors argued for ‘a dramatic shift in priorities’ and ‘major investment in a system of comprehensive maternity care’, calling upon the World Bank to prioritise maternal mortality and take the lead in building and funding a preventive health programme [36]. Also in 1985, during a conference marking the end of the UN Decade for Women, a WHO speaker announced the same estimate of half a million annual maternal deaths [37]. In response to increasing concern and demand for data, WHO published 1983 data in 1986 [38] and began monitoring trends, continually improving the modelling and estimation methods, and publishing reports every few years (see Table 2).

**Table 2:** Global estimates of MMR and lifetime risk of maternal death produced by WHO and partner agencies, published from 1985 to 2019

Tabulations of available information by the World Health Organization (WHO)	
Publication date and title	Description and key reported estimates
1985: *Maternal mortality rates: a tabulation of available information* (unpublished)	This report was compiled and informally disseminated (but not published) by WHO, in response to many requests for information.
1986: *Maternal mortality rates: a tabulation of available information, second edition* [38]	Estimates for 1983: Maternal deaths in 1983/annual: 500,000 (99% of these in developing countries, where 86% of births take place). MMR: World: 390 Developing countries: 450 Developed countries: 30 Region with highest MMR: Western Africa, 700
1991: *Maternal mortality ratios and rates: a tabulation of available information, third edition* [39]*	Estimates for 1988: Maternal deaths in 1988: 509,000 MMR: World: 370 (lifetime risk of maternal death [LR]: 1 in 73) Developing countries: 420 (LR: 1 in 57) Developed countries: 26 (LR: 1 in 1825) Region with highest MMR: Western Africa, 760 (LR 1 in 18)
WHO and UNICEF collaboration to develop revised 1990 estimates using a new approach	
1996: *Revised 1990 estimates of maternal mortality: a new approach by WHO and UNICEF* [40]†	Estimates for 1990: Maternal deaths in 1990: 585,000 MMR: World: 430 (LR: 1 in 60) Less developed regions: 480 (LR: 1 in 48) More developed regions: 27 (LR: 1 in 1800) Region with highest MMR: Eastern Africa, 1060 (LR: 1 in 12)
Series of estimates of maternal mortality developed by WHO and UN partners	
2001: *Maternal mortality in 1995: estimates developed by WHO, UNICEF, UNFPA* [41]	Estimates for 1995: Maternal deaths in 1995: 515,000 World: 400 (LR: 1 in 75) More developed countries: 21 (LR: 1 in 2500) Less developed countries: 440 (LR: 1 in 60) Least developed countries: 1000 (LR: 1 in 16) Region with the highest MMR: Eastern Africa, 1300 (LR: 1 in 11)
2004: *Maternal mortality in 2000: estimates developed by WHO, UNICEF, UNFPA* [42]	Estimates for 2000: Maternal deaths in 2000: 529,000 MMR: World: 400 (LR: 1 in 74) Developing regions: 440 (LR: 1 in 61) Developed regions: 20 (LR: 1 in 2800) Region with the highest MMR: Sub-Saharan Africa, 920 (LR: 1 in 16)
2007: *Maternal mortality in 2005: estimates developed by WHO, UNICEF, UNFPA and The World Bank* [43]	Estimates for 2005: Maternal deaths in 2005: 536,000 (99% in developing countries) MMR: World: 400 (LR: 1 in 92) Developing regions: 450 (LR: 1 in 75) Developed regions: 9 (LR: 1 in 7300) Region with highest MMR: Sub-Saharan Africa, 900 (LR: 1 in 22)
Series of estimates and trends of maternal mortality developed by the UN’s Maternal Mortality Estimation Inter-Agency Group (UN MMEIG) – WHO, UNICEF, UNFPA, World Bank Group and UNPD*	
2010: *Trends in maternal mortality: 1990 to 2008: estimates developed by WHO, UNICEF, UNFPA and The World Bank* [44]	Estimates for 2008, and revised estimates for 1990, 1995, 2000, 2005 Maternal deaths in 2008: 358,000 MMR in 2008: World: 260 (LR: 1 in 140) Developing regions: 290 (LR: 1 in 120) Developed regions: 14 (LR: 1 in 4300)
This and later reports use UN MDG regions	Region with the highest MMR: Sub-Saharan Africa, 640 (LR: 1 in 31)
2012: *Trends in maternal mortality: 1990 to 2010: WHO, UNICEF, UNFPA and The World Bank estimates* [45]	Estimates for 2010, and revised estimates for 1990, 1995, 2000, 2005 Maternal deaths in 2010: 287,000 MMR in 2010: World: 210 (LR: 1 in 180) Developing regions: 240 (LR: 1 in 150) Developed regions: 16 (LR: 1 in 3800) Region with the highest MMR: Sub-Saharan Africa, 500 (LR: 1 in 39)
2014: *Trends in maternal mortality: 1990 to 2013: estimates by WHO, UNICEF, UNFPA, The World Bank and the United Nations Population Division* [46]	Estimates for 2013, and revised estimates for 1990, 1995, 2000, 2005, 2010 Maternal deaths in 2013: 289,000 MMR in 2013: World: 210 (LR: 1 in 190) Developing regions: 230 (LR: 1 in 160) Developed regions: 16 (LR: 1 in 3700) Region with the highest MMR: Sub-Saharan Africa, 510 (LR: 1 in 38)
2015: *Trends in maternal mortality: 1990 to 2015: estimates by WHO, UNICEF, UNFPA, World Bank Group and the United Nations Population Division* [20]	Estimates for 2015, and revised estimates for 1990, 1995, 2000, 2005, 2010 (see Table 3) Maternal deaths in 2015: 303,000 MMR in 2015:‡ World: 216 (LR: 1 in 180) Developing regions: 239 (LR: 1 in 150) Developed regions: 12 (LR: 1 in 4900) Region with the highest MMR: Sub-Saharan Africa, 546 (LR: 1 in 36)
2019: *Trends in maternal mortality: 2000 to 2017: estimates by WHO, UNICEF, UNFPA, World Bank Group and the United Nations Population Division* [1]	Estimates for 2017, and revised estimates for 2000, 2005, 2010, 2015 Maternal deaths in 2017: 295,000 MMR in 2017: World: 211 (LR: 1 in 190) Less developed regions: 232 (LR: 1 in 160) More developed regions: 12 (LR: 1 in 5200)

Data from each report cannot be meaningfully compared to data from previous reports/years due to subsequent advances in modelling/estimation methods for each publication. The UN MMEIG was formed in 2010. The estimates presented in each *Trends in maternal mortality* report listed above supersede all previously published estimates for years that fall within the same time period due to modifications in methodology and data availability. Therefore, differences between newer and previous estimates should not be interpreted as representing time trends; trends should only be interpreted as presented within each separate publication for the years covered [1].

* In addition to the reports of estimates and trends in maternal mortality, WHO also published *Maternal mortality: a global factbook* in 1991, presenting 1983 MMR estimates as well as 1985 estimates for coverage of maternity care (per cent of births with a trained attendant), country profiles compiling all available demographic and maternal health information, and detailed information about measuring maternal mortality [47].

† These revised 1990 estimates were required because the model used to calculate previous 1990 estimates could not support accurate country-level estimates but just regional and global estimates. The earlier 1990 estimates were inadvertently published in the 1992 *Human Development Report* but were never used.

‡ Unfortunately, these estimates were not available before the 2015 MDG report was published in the same year [26], so that report used the 1990 baseline and 2013 MMR estimates from the previous (2014) UN MMEIG publication.

The culmination of this gathering momentum – when maternal mortality achieved high-priority status – was the **Safe Motherhood Conference** in 1987 in Nairobi, Kenya, co-sponsored by the World Bank, WHO and UNFPA. At the event, WHO’s Director General acknowledged that maternal mortality had indeed been a neglected tragedy, ‘because those who suffer it are neglected people, with the least power and influence over how national resources shall be spent’ [48]. The Safe Motherhood Initiative (SMI) was launched, including a plan for halving maternal mortality by 2000, and calling for cost-effective health services delivered through a three-tiered system: village/community level; hospital/district level; and an ‘alarm and transport’ system to transfer women to appropriate facilities [49]. Reportedly, many conference participants ‘noted that reliable data on maternal mortality and morbidity in developing countries are either unavailable or incomplete and agreed that there is an urgent need to develop appropriate record-keeping systems at all levels’, to help countries monitor and improve women’s health [49]. Appendix 1 provides summary information on population-based surveys reporting on maternal, newborn and child health (MNCH) since the 1970s, and Appendix 2 briefly describes the particular challenges and methods of measuring and monitoring maternal mortality.

After the conference, the Safe Motherhood Inter-Agency Group (IAG) was established by the World Bank, WHO, UNFPA, UNICEF, the United Nations Development Programme (UNDP), International Planned Parenthood Federation (IPPF) and the Population Council [37,50] as a forum for continued collaboration to achieve the SMI goals. In 2004, the IAG evolved to become the Partnership for Safe Motherhood and Newborn Health.

In the spirit of the Alma-Ata Declaration, the main interventions prioritised under the SMI were community-based preventive-care interventions, including screening pregnant women for risks of complications and training traditional birth attendants to improve community-level delivery care. Both of these approaches were dropped after it was acknowledged at the 10th anniversary conference in 1997 that they had not been effective at reducing maternal mortality; the new messages were ‘every pregnancy faces risks’ and ‘ensure skilled attendance at delivery’. Focus on safe motherhood became diffused in efforts to address the plethora of contributing factors facing girls and women, such as early marriage, lack of access to education and contraception, and poor nutritional, socio-economic and legal status [37]. Certainly, the SMI goal of halving maternal mortality by 2000 was not reached: global MMR fell 11% from 385 to 341 maternal deaths per 100,000 live births between 1990 and 2000 (see Table 3) [20]. Nevertheless, the conference kick-started a process of raising global awareness, developing and implementing effective interventions, and improving measurement approaches.

**Table 3:** Trends in United Nations estimates of maternal mortality ratios (MMR), neonatal mortality rates (NMR) and stillbirth rates (SBR), during the MDG era: 1990–2015

Region	1990	1995	2000	2005	2010	2015	% change 1990–2015
MMR*							
World	385	369	341	288	246	216	44
Developed regions	23	22	17	15	13	12	48
Developing regions	430	409	377	319	273	239	44
NMR							
World†	37	34	31	26	22	19	49
Developed regions‡	8	NA	NA	NA	NA	3	58
Developing regions‡	40	NA	NA	NA	NA	21	47
SBR§							% change 2000–2015
World	NA	NA	24.7	NA	NA	18.4	25.5
Developed regions	NA	NA	4.5	NA	NA	3.4	24.4
Sub-Saharan Africa	NA	NA	35.6	NA	NA	28.7	19.4

* WHO 2015 [20] by UN MDG region.

† UNIGME 2018 [51] by SDG region.

‡ UNIGME 2015 [27] by MDG region (only available for 1990 and 2015).

§ Blencowe.

### Key conferences and goals set in the 1990s: paving the way to the MDGs

Three major global conferences in the 1990s built on the momentum and concern surrounding maternal mortality after the Safe Motherhood Conference. They also reiterated and/or expanded upon the SMI’s main goal of halving maternal mortality by 2000. First was the **1990 World Summit for Children**, in New York City. The first two major goals of the ‘World Declaration on the Survival, Protection and Development of Children’ were to reduce the infant mortality rate (IMR) and U5MR by one-third, and to reduce the MMR by half by 2000 [52].

Next, in 1994, the UN’s third and final official world conference on population was held in Cairo, the **International Conference on Population and Development (ICPD)**. The ICPD’s widely lauded Programme of Action (PoA) – as Ban Ki-moon later stated – ‘put people’s rights at the heart of development’ and ‘affirmed sexual and reproductive health as a fundamental human right’ [11]. The World Summit for Children’s targets for reducing IMR, U5MR and MMR by 2000 were adopted, and targets for 2015 were added. Targets for providing universal access to reproductive health (RH) services were also set. The PoA was adopted by 179 countries, and its MCH and RH action points and targets directly laid the groundwork for MDGs 4 and 5 set in 2000 (see MDG targets in Table 1 and ICPD objectives/targets in Box 3). No targets were set for neonatal mortality, morbidity or stillbirths (the same was true of the World Summit for Children), but the connection between child survival and maternal RH was noted (and a definition of RH was presented; see Box 1). The wide gap between maternal mortality in higher- and lower-income regions was a critical part of the ‘basis for action’, and emphasis was also placed on ‘adequate evaluation and monitoring’ to assess progress and enhance programme effectiveness [11].

Box 3 ICPD objectives and targets for improvement of maternal and child health, from the PoA section on ‘Health, morbidity and mortality’

**Child survival and health**

Objectives:

(a) To promote child health and survival and to reduce disparities between and within developed and developing countries as quickly as possible, with particular attention to eliminating the pattern of excess and preventable mortality among girl infants and children;

*(b) To improve the health and nutritional status of infants and children*. (c) To promote breastfeeding as a child survival strategy.

Relevant action points/targets:

- ‘Countries should strive to reduce their infant and under-five mortality rates by one-third, or to 50 and 70 per 1,000 live births, respectively, whichever is less, by the year 2000, with appropriate adaptation to the particular situation of each country’.
- ‘By 2015, all countries should aim to achieve an infant mortality rate below 35 per 1,000 live births and an under-five mortality rate below 45 per 1,000’.

**Women’s health and safe motherhood**

Objectives:

(a) To promote women’s health and safe motherhood; to achieve a rapid and substantial reduction in maternal morbidity and mortality and reduce the differences observed between developing and developed countries and within countries. On the basis of a commitment to women’s health and well-being, to reduce greatly the number of deaths and morbidity from unsafe abortion;

(b) To improve the health and nutritional status of women, especially of pregnant and nursing women.

Relevant action points/targets:

- ‘Countries should strive to effect significant reductions in maternal mortality by the year 2015: a reduction in maternal mortality by one half of the 1990 levels by the year 2000 and a further one half by 2015’.
- ‘All births should be assisted by trained persons, preferably nurses and midwives, but at least trained birth attendants’.

**Reproductive rights and reproductive health**

Objectives:

(a) To ensure that comprehensive and factual information and a full range of reproductive healthcare services, including family planning, are accessible, affordable, acceptable and convenient to all users;

(b) To enable and support responsible voluntary decisions about childbearing and methods of family planning of their choice, as well as other methods of their choice for regulation of fertility which are not against the law and to have the information, education and means to do so;

(c) To meet changing reproductive health needs over the life cycle and to do so in ways sensitive to the diversity of circumstances of local communities.

Relevant action points/targets:

- ‘All countries should strive to make accessible through the primary healthcare system, reproductive health to all individuals of appropriate ages as soon as possible and no later than the year 2015’.

Source: United Nations 2014 [11]

Just a year later, the Declaration issued by the **1995 Fourth World Conference on Women**, in Beijing, included the following point: ‘The explicit recognition and reaffirmation of the right of all women to control all aspects of their health, in particular their own fertility, is basic to their empowerment’ [53]. An agreed action point was to ‘reduce ill health and maternal morbidity’. The Beijing Declaration restated the 2000 and 2015 targets for reducing IMR, U5MR and MMR set at the ICPD (see Box 3) [53], adding further weight to these goals.

### Improved monitoring of obstetric services to reduce maternal mortality

In 1997, in direct response to the stated challenge to halve MMR by 2000 and reduce it by three quarters by 2015 – targets which had already been mentioned and endorsed in multiple international initiatives and publications – UNICEF, WHO and UNFPA issued *Guidelines for monitoring the availability and use of obstetric services*. This publication aimed to support countries to regularly monitor progress by assessing the quality and coverage of interventions aimed at improving emergency obstetric services to reduce maternal mortality; it presented revised process indicators, and guidance and tools for data collection and analysis [54]. In 2009, an updated handbook was published [55] a collaborative effort by WHO, UNFPA, UNICEF and Averting Maternal Death and Disability (AMDD).

### The MDG era: setting targets for 2015 and tracking progress

In September 2000, world leaders came together at the UN in New York for the Millennium Summit to adopt the **UN Millennium Declaration**, committing their nations to a new global partnership to eradicate extreme poverty, and setting out a series of goals to be achieved by 2015, which soon came to be known as the MDGs. The Declaration, signed by 189 countries, included the resolution ‘to have reduced maternal mortality by three quarters, and under-five child mortality by two-thirds, of their current rates’ by 2015 [56], formalising similar targets set at the ICPD.

When the wording of all eight MDGs was later finalised, with associated targets and indicators for each, MDG 5 – ‘Improve maternal health’ – included target 5.A: ‘Reduce by three quarters, between 1990 and 2015, the maternal mortality ratio (MMR)’. There was no target addressing maternal morbidity, but target 5.B – ‘Achieve, by 2015, universal access to reproductive health’ – was officially added to the MDGs in 2006 after the importance of this goal – originally set at the ICPD – was reaffirmed by leaders at the 2005 UN World Summit [57]. MDG 4 was ‘Reduce child mortality’, and target 4.A was ‘Reduce by two-thirds, between 1990 and 2015, the under-five mortality rate’ (see Table 1). No targets or indicators were specified for neonatal mortality or morbidity, or for stillbirths.

In the decade leading up to the MDG era – when health and development goals to be measured against the 1990 level were already gaining traction – and in the decade after the launch of the MDGs – when 1990 had been fixed as the official baseline – there was a major drive to develop accurate global, regional and national estimates for the year 1990. With these efforts to establish the baseline data and monitor subsequent progress, the MDG era prompted improvements in data collection and analysis methods for maternal and child mortality. The MMR estimates proved especially challenging and have been revised frequently; the final UN data for the MDG era were published in 2015 [20], and new estimates for 2000–2017 have been published in 2019 [1]. Efforts to improve the measurement of MMR were intensified in the last five years of the MDG era with the formation in 2010 of the **UN’s Maternal Mortality Estimation Inter-Agency Group (UN MMEIG)** – comprising WHO, UNICEF, UNFPA, World Bank Group and UNPD – to harmonise various UN estimates and improve estimation and modelling methods [58] (see the final section of Table 2, and Appendix 2).

In parallel to estimation work of the UN MMEIG, the Institute for Health Metrics and Evaluation (IHME) at the University of Washington in Seattle, USA, has also published a number of reports of global, regional and national estimates of maternal mortality (number of deaths and MMRs) since 2010. Both groups use model-based estimates, but use different modelling approaches including the model specification, selection of covariates and approach to addressing HIV-related deaths. In addition, the UN MMEIG engages countries in an official WHO country consultation process when the preliminary estimates have been derived, to discuss methods and data inputs before the global, regional and national data are finalised and published. More details about the estimates generated by the different entities can be found in a 2011 publication by AbouZahr [59].

The **‘Countdown to 2015’** collaboration of UN agencies, implementing partners and academics was established in 2003 to support monitoring of the coverage of interventions needed to reduce maternal and child mortality and to promote accountability from governments and partners working to provide equitable coverage of these interventions [60,61]. Progress reports for stake-holders were issued every two to three years. While originally focusing on newborn and child survival [62], subsequent reports encompassed the MNCH continuum of care, expanding the selection criteria used for the priority countries, such that the final 2015 report covered 75 priority countries, which accounted for more than 95% of all maternal, newborn and child deaths [63]. The Countdown ‘country profiles’ were hailed as a key achievement for tracking country-level progress towards the MDGs.

The MDG era also sparked other key global initiatives such as **Women Deliver**, an organisation focused on gender equality and girls’ and women’s health and rights (the first Women Deliver conference was held in London in 2007, marking the 20th anniversary of the Safe Motherhood Conference), and the 2008 launch of the **Maternal Health Task Force** (MHTF) at the Harvard Chan School of Public Health.

### Neonatal mortality: slowly gaining prominence in the global health agenda

Although none of the major conferences in the 1990s included a focus on neonatal mortality, morbidity or stillbirths, there were some efforts to monitor these outcomes. In 1996, WHO published available data on perinatal mortality [64], a decade after first publishing available MMR data [38]. The report estimated almost 4.3 million foetal deaths and more than 5 million neonatal deaths in 1995, recommending that ‘To reduce infant deaths substantially, the focus will have to be on reducing neonatal deaths, particularly in the early neonatal period when more than four out of 10 infant deaths and most neonatal deaths occur’ [64]. Despite this timely publication and recommendation, no targets for neonatal mortality or stillbirths were included in the MDGs.

Key publications and events in 2005 raised the profile of the burden of neonatal mortality. In March, *The Lancet* Neonatal Survival Series drew attention to a ‘crucial omission in global health research and policy’ [65]. The series culminated in a call to focus on improving neonatal survival to meet MDG target 4.A on U5MR, and to add a target to reduce neonatal mortality [66]. Weeks later, WHO’s *World Health Report 2005* – *Make every mother and child count* reported ‘patchy progress and widening gaps’ and included a chapter titled ‘Newborns: no longer going unnoticed’ [67]. In May 2005, a **WHA resolution** was issued for universal coverage of MNCH interventions, putting maternal, newborn and child health and survival together as the top priority for ministers of health [62]. It urged Member States ‘to establish or sustain national and international targets, and to establish monitoring mechanisms for measuring progress towards the achievement of agreed goals’ [68]. By this time, the familiar abbreviation ‘MCH’ had evolved into ‘MNCH’ – placing newborns firmly into the mix. Groups focusing on maternal and/or child health began to embrace issues of newborn survival. In September 2005, the **Partnership for Maternal, Newborn and Child Health (PMNCH)** was launched, bringing together three existing partnerships – the Partnership for Safe Motherhood and Newborn Health, the Healthy Newborn Partnership and the Child Survival Partnership – to support the achievement of MDGs 4 and 5 [69]. PMNCH emphasises the importance of providing services along the full continuum of care for women and children; it now includes more than 1000 partners in 77 countries [69].

Soon after this surge of activity in 2005 relating to newborn survival, in 2006, WHO published data on global, regional and national neonatal and perinatal mortality and stillbirth rates for 2000 [70], and then published data for 2004 a year later, ‘in response to a surge in national community studies and acknowledging improved reporting of vital registration data’ [71]. The 2004 data indicated a global NMR of 28 per 1000 live births, with shocking disparity between higher- and low-income countries: 4 vs 41.

The **UN Inter-Agency Group for Child Mortality Estimation (UN IGME)**, led by UNICEF and WHO, was formed in 2004 to improve, share and harmonise data on child mortality [72]. Since 2010, the group has published annual reports on ‘Levels and trends in child mortality’, using data from their collaborative research initiatives and national household surveys, such as the Demographic and Health Surveys (DHS) and the Multiple Indicator Cluster Surveys (MICS) (see Appendix 1). The group tabulated NMRs for the first time in 2011, noting that ‘over the last two decades almost all regions have seen slower declines in neonatal mortality than in under-five mortality’ [73]. Their 2018 report presents the estimates for 2017, and five-yearly estimates since 1990 [51] and the latest neonatal mortality data for 1990–2018 have been released in September 2019 [2] (see global data in Table 3).

To focus attention on newborn health, in 2008 Saving Newborn Lives (SNL; an initiative of Save the Children) convened the **Newborn Indicators Technical Working Group (TWG)** with representatives from SNL, UNICEF, USAID, DHS and other implementing partners. The TWG was established to assess and develop standardised indicators to monitor and evaluate newborn health – indicators that can be tracked via population-based surveys (e.g. DHS and MICS), independent studies and routine health information systems [74].

Between 2010 and 2015, there was a proliferation of global initiatives with an explicit focus on newborns, beginning with the Muskoka Initiative for MNCH, a five-year major funding commitment agreed at a G-8 Summit in 2010 [75]. In 2012, the Child Survival Call to Action was convened jointly by UNICEF and the governments of Ethiopia, India and the USA, leading to the launch of A Promise Renewed – ‘a global effort to accelerate action on maternal, newborn and child survival’ [76]. More than 178 governments and 600 civil society and private sector organisations pledged support [77]. Then in April 2013, the first **Global Newborn Health Conference** was held in Johannesburg, with participants from more than 50 countries [78]. The conference included preliminary consultation on the development of a global newborn action plan and a common monitoring framework. This kicked off a series of further consultations [79], culminating in the launch of the **Every Newborn Action Plan (ENAP)** in 2014 [24] (ENAP is discussed below). Also in 2014, *The Lancet* published the Every Newborn Series, including data and trends on neonatal mortality and proposing national and global targets; the authors emphasised that ‘To count deaths is crucial to change them’ [80]. In 2015, the ENAP target for reduction of NMR by 2030 was included in the SDGs (see Table 1) and in the Global Strategy for Women’s, Children’s and Adolescents’ Health [81] (see section on The Global Strategy).

But why did prioritisation of newborn survival lag so far behind that of maternal survival, despite the much larger number of annual neonatal deaths, and the fact that both groups face the highest risks during the perinatal period, when women and babies could both be reached with joined-up interventions? According to the conclusions of a 2016 study on this subject, which examined the emergence and growth of political priority for these issues, maternal survival was strongly positioned as a social justice issue in the 1980s, drawing attention and resources since then, while newborn survival came under the umbrella of ‘maternal and child survival’ but was not fully adopted by either the ‘maternal’ or ‘child’ camp. Smith and Shiffman concluded that ‘network expansion and alignment with child survival norms have improved the issue’s status in the past few years’ [82].

### Stillbirths: bringing them into the fold

We have seen that there was substantial momentum for better monitoring and faster reduction of neonatal mortality from around 2005 to 2015, when a target for reduction made it into the SDGs. But it took longer for stillbirths to be addressed seriously and no SDG target was set.

In 2006, both WHO [70] and Stanton *et al*. [83] had produced very similar estimates of stillbirth rates (SBR) for 2000, while acknowledging the lack of reliable data in many countries, and the next year, WHO released updated estimates for 2004 [71]. A few years later, in 2009, six new reviews on reducing stillbirths, with a focus on LMICs, were published in a supplement of *BMC Pregnancy and Childbirth*. The issue highlighted that most stillbirths in LMICs are late pre-term, term and intrapartum, which are relatively easy to prevent [84]. Lawn *et al*. reviewed the global epidemiology and causes of stillbirths as well as the availability and quality of data, while acknowledging that the relative ‘invisibility’ of stillbirths on the global agenda was due to ‘a lack of data and a lack of consensus on priority interventions, but also to social taboos that reduce the visibility of stillbirths and the associated family mourning’ [85]. In 2011, *The Lancet* Stillbirths Series examined the data and available interventions, including an analysis by Cousens *et al*. presenting SBR estimates for 2009 and trends since 1995. The authors noted the ‘dearth of reliable data in regions where most stillbirths occur’ and that SBR was declining more slowly than MMR and U5MR [86]. Another article in the series called for the inclusion of stillbirth in relevant international health reports and initiatives, and for clear targets [87].

SBRs were included for the first time in the ‘Count-down to 2015’ country profiles in 2010, based on improved availability of data and evidence of the close links with maternal and newborn health [63]. In 2014, in anticipation of the setting of new global goals, the Every Newborn Action Plan (ENAP) proposed targets for reducing NMR and SBR (see Box 4, and section below on ENAP) [24], but despite this and other efforts to bring stillbirths more firmly onto the global agenda at that time [88], no target for reducing stillbirths was included in the SDGs in 2015. Nevertheless, the 2016 *Indicator and monitoring framework for the Global Strategy for Women’s, Children’s and Adolescents’ Health (2016*–*2030)* (see below) included SBR as an additional indicator linked to SDG target 3.2.2 on neonatal mortality, and selected it as one of 16 key indicators, along with MMR, U5MR and NMR [15]. In 2016, *The Lancet* Ending Preventable Stillbirths Series provided new data and reviewed progress [3,89], and a systematic analysis of national, regional and global SBR estimates from 2000 to 2015 was published in *The Lancet Global Health* [12]. All of this has put greater focus on stillbirths at this crucial time when more action is clearly needed.

Box 4 Every Newborn Action Plan (ENAP) and Ending Preventable Maternal Mortality (EPMM) goals and targets

**ENAP goals**

1. By 2035, all countries will reach the target of 10 or less newborn deaths per 1000 live births and continue to reduce death and disability, ensuring that no newborn is left behind. Achievement of this target will result in an average global neonatal mortality rate (NMR) of 7 deaths per 1000 live births.
  - By 2030, all countries will reach 12 of less newborn deaths per 1000 live births resulting in an average global NMR of 9 deaths per 1000 live births.
2. By 2035, all countries will reach the target of 10 or less stillbirths per 1000 total births and continue to close equity gaps. Achieving this goal will result in an average global stillbirth rate (SBR) of 8 per 1000 total births.
  - By 2030, all countries will reach 12 or less stillbirths per 1000 total births, resulting in an average global SBR of 9 deaths per 1000 total births.

These goals are supported by a set of six guiding principles and five strategic objectives, as well as specific, evidence-based interim coverage targets for 2020 and 2025, and global and national milestones to be reached by 2020.

Source: WHO 2014 [24].

**EPMM targets**

- **Global target *to increase equity in maternal mortality between countries***: By 2030, all countries should reduce maternal mortality ratio (MMR) by at least two-thirds of their 2010 baseline level. The average global target is an MMR of less than 70 maternal deaths per 100 000 live births by 2030.
- **Supplementary national target:** By 2030, no country should have an MMR greater than 140, a number twice the global target.

Country targets for 2030 were also set, depending on baseline levels of MMR, to increase equity in maternal mortality, since the global target may not be applicable to individual countries.

Source: WHO 2015 [90].

### The Global Strategy: final push on MDGs 4 and 5

The UN Secretary-General’s **Global Strategy for Women’s and Children’s Health** was launched at the 2010 UN Summit on the MDGs, to hasten progress towards MDGs 4 and 5. Key areas for action included support for country-led plans, and ‘improved monitoring and evaluation to ensure the accountability of all actors for results’ [91]. **Every Woman Every Child** (EWEC) was launched at the same time – a global movement to enact the Global Strategy’s roadmap, by mobilising and intensifying national and international action and resources [92].

After the launch, the UN Secretary-General called on WHO to ensure ‘the most effective international institutional arrangements for ensuring global reporting, oversight and accountability’. In response, the **Commission on Information and Accountability for Women’s and Children’s Health (CoIA)** was established. Its report, published in 2011, proposed an accountability framework for women’s and children’s health which ‘covers national and global levels and comprises three interconnected processes – monitor, review and act’ [93]. MMR and U5MR topped the list of indicators selected for monitoring the status of women’s and children’s health, along with stunting in children under 5, and eight coverage indicators. The first three of the 10 CoIA recommendations concerned ‘better information for better results’, relating to improved vital events registration, use of common indicators, and innovative use of information and communications technologies in national health systems.

CoIA’s final recommendation was to establish an independent Expert Review Group (iERG) on global reporting to operate until 2015 [93]. In its final report, the iERG concluded that between 2010 and 2015, while the intention had been to save 16 million lives by achieving MDGs 4 and 5, in fact just 2.4 million lives were saved [94]. The next challenges were identified as follows: ‘im-proving the quality of information available for delivering accountability, obtaining political commitment, ensuring regular reporting and strengthening civil society engagement’ [94].

At the end of the MDG era, the Global Strategy was updated to align with the priorities and timeline (2016– 2030) of the SDGs, and adolescents were added to the focus. The revised objectives are ‘survive, thrive and transform’; in other words, ‘to end preventable mortality and enable women, children and adolescents to enjoy good health while playing a full role in contributing to transformative change and sustainable development [81]. The indicator and monitoring framework for the Global Strategy, published in 2016, is structured around the relevant SDG 3 targets and their 34 indicators, but with 26 ‘additional indicators’ (including SBR) drawn from existing global initiatives [15], such as from ENAP and EPMM (see below).

## The Every Newborn Action Plan (ENAP) and Ending Preventable Maternal Mortality (EPMM): convergent initiatives to guide the SDG agenda on MNCH

### ENAP – to end preventable stillbirths and newborn deaths

In May 2014, the WHA endorsed a resolution for a ‘newborn health action plan’ [95], and WHO subsequently launched ENAP, presenting actions and targets for saving the lives of nearly 3 million babies and women every year [24]. ENAP acknowledged that newborn deaths and stillbirths had so far received inadequate attention and investment, and called for improved ‘access to, and quality of, health care for women and newborns within the continuum of care’, building upon the Global Strategy and EWEC. ENAP set goals for NMR and SBR reductions by 2030 and 2035 (see Box 4) [24], but while the NMR target was adopted as an SDG target, the SBR target was not. One of the five ENAP strategic objectives is ‘Count every newborn through measurement, programme-tracking and accountability’ [24], underlining ‘the urgent need for improved national data’ [96]. The ENAP Metrics Working Group was formed in September 2014, co-chaired by WHO and the London School of Hygiene & Tropical Medicine, to work with countries and partners to meet the ENAP milestones and widely disseminate tools and learning [97]. The ENAP Measurement Improvement Roadmap was developed in 2015 to improve data collection by 2020. It outlined tools to be developed and ‘actions to test, validate and institution-alise proposed coverage indicators’, working with existing stakeholders to strengthen health information and civil registration and vital statistics (CRVS) systems [98]. ‘Ser-vice readiness for the small and sick newborn’ has been identified as a major measurement gap, and there is ongoing work to address this and improve the quality of care for these newborns [96].

### EPMM – targets and strategies

Similar to the ENAP initiative and also in anticipation of the launch of the SDGs, WHO facilitated the development of a strategy paper on EPMM in 2015 [90], in collaboration with UNICEF, UNFPA, USAID and other partners. The EPMM strategy focuses on maternal mortality but encourages linkages with ENAP to address the MNCH continuum of care [90]. One of the cross-cutting actions called for is to ‘improve metrics, measurement systems and data quality’ to ensure that all maternal and newborn deaths are counted, through effective national surveillance and CRVS systems in every country [90]. As tools for this, the EPMM strategy points to standard definitions for causes of death found in the International Classification of Diseases (ICD), together with WHO guidance on their application [4], as well as use of Maternal Death Surveil-lance and Response (MDSR) systems, perinatal death surveillance, confidential enquiries and other data sources (see Appendix 2). EPMM proposed new ‘ambitious yet feasible’ targets for 2030 (see Box 4) – the MMR target was adopted as SDG target 3.1 – and a monitoring framework was developed [99,100].

### Convergence and merging of ENAP and EPMM and MNCH initiatives

The ENAP Metrics Working Group and the EPMM Working Group merged in 2015 to harmonise their targets, indicators and other considerations, in line with the Global Strategy and EWEC (see previous section), creating the joint indicator and monitoring framework for the Global Strategy [15]. In addition, in 2015 WHO launched **MoNITOR (Maternal and Newborn Information Tracking for Outcomes and Results)** – a technical advisory group of 14 independent global experts – ‘to ensure harmonised guidance, messages, and tools so that countries can collect useful data to track progress towards achieving the Sustainable Development Goals’[101]. MoNITOR is in the process of publishing recommendations on priority indicators for monitoring maternal and newborn health (MNH) [102]. Also in 2015, the **Global Maternal Newborn Health Conference** was held in Mexico City, bringing together partners working on the implementation and monitoring of ENAP, EPMM and other MNH initiatives, ‘to share new evidence, identify opportunities and gaps, build understanding across disciplinary boundaries, and discuss the way forward to improve maternal and newborn health around the globe’ [103].

## Monitoring maternal and neonatal morbidity: the missing pieces

This paper has so far examined the challenging process of getting maternal mortality, neonatal mortality and finally stillbirths onto the global health and development agenda. Similar efforts to raise the profile of maternal and neonatal morbidity with improved monitoring remain at a nascent stage. There are no internationally agreed and tracked indicators(potential candidates are listed in Box 2), but research is ongoing. For some perspective on these efforts, we need to step back, to around the turn of the millennium.

### Maternal morbidity

In 2004, WHO conducted a systematic review of the incidence and prevalence of maternal mortality and morbidity [104]. Although thousands of articles were retrieved, the reporting quality was generally low and methodological challenges hindered the review process. Another systematic review that year by some of the same authors investigated the prevalence of severe acute maternal morbidity (SAMM, also termed ‘near miss’), but the need for uniform criteria presented a major challenge [105].

Faced with these difficulties, WHO established a working group to develop a standard definition of and uniform criteria for ‘maternal near miss’ (MNM). In 2009, the group published their definition of MNM (see Box 1) and proposed case-identification criteria and MNM-based indicators for monitoring quality of obstetric care [6]. Given the interest in MNM as an adjunct to maternal mortality, a systematic review and analysis of the prevalence of MNM, based on the new definition, were published in 2012 [106]. The results of the meta-analysis yielded MNM prevalence estimates of 0.42% for organ dysfunction and 0.039% for emergency hysterectomy.

WHO then initiated a five-year project (2012–2017), starting with the formation of the Maternal Morbidity Working Group (MMWG) of global experts. The group worked to systematically to explore the meaning of maternal morbidity and ‘examined in depth how best to define, describe, and measure it for the purposes of research, epidemiology, and ultimately to improve women’s experience of the care they receive’ [107]. In 2016, the group published a definition of maternal morbidity (see Box 1) and a matrix of 121 conditions [5], and in 2018 published a special supplement of the *International Journal of Gynecology & Obstetrics* [108], describing the formative findings of the project and how maternal morbidity is necessarily reconceptualised to describe the experiences of women, and the implications for health systems and policy.

In a systematic review of systematic reviews by Gon *et al*. in the 2018 special issue, for 71% of the 121 listed morbidities, no systematic review was found, including for some very serious conditions. Based on the available data, global estimates were presented for direct maternal morbidities, including post-partum haemorrhage 6–11%, preeclampsia 2.3%, severe complications of unsafe abortion 0.6%, eclampsia 0.5% and regional estimates for gestational diabetes mellitus (5.1% in Africa and 25.1% in the Western Pacific Region). Estimates of indirect maternal morbidities included obstetric fistula in LMICs 0–1.6% of post-partum women, post-partum depression in LMICs 1–50%, anxiety during pregnancy 4.4–39% worldwide, post-partum anxiety 8.5% worldwide, pooled HIV incidence rate in sub-Saharan Africa at 4.7 per 100 person-years during pregnancy and 2.9 per 100 person-years during the post-partum period, syphilis in pregnancy in LMICs 0.5-8.3%, chlamydia in pregnancy in LMICs 0.4–16.4%, malaria during pregnancy 29.5% in East and Central Africa and 35.1% in West and Central Africa, and a median of 4.3% of pregnancies are diagnosed with seroprevalence of hepatitis B serum antigen (HBsAg), and between 2.5% and 3.0% of pregnant women in Africa are infected with hepatitis C [109].

The **Child Health Epidemiology Reference Group (CHERG)**, a group of independent technical experts established by WHO in 2001 (now known as the **Maternal and Child Epidemiology Estimation Group [MCEE]**), undertook research on maternal morbidity, mainly to estimate the burden of related diseases and sequelae. They noted that the causal pattern of morbidity is different from that of mortality, and that information on morbidity could have implications for the prioritisation of safer motherhood interventions [110].

There is still much work to be done before maternal morbidity can be accurately measured and monitored, and clear global or national targets set for its reduction. Currently, MNM cases and MNM ratios are not widely reported (see Box 2).

### Neonatal morbidity

There is as yet no agreed definition or global indicator for neonatal morbidity, although this was a priority area as identified in the ENAP Measurement Improvement Roadmap [98]. Low birthweight (LBW: < 2500 g; see Box 2) has been used as a marker for babies at highest risk for neonatal morbidity, as described by Lawn *et al*. [80]. LBW or small size at birth ‘is the biggest risk factor for more than 80% of neonatal deaths and increases risk of post-neonatal mortality, growth failure, and adult-onset non-communicable diseases’ and small babies also face the highest risk of death in utero [80]. Further available information on neonatal morbidities is provided in that report.

In 2002, the United Nations General Assembly Special Session on Children adopted the goal of reducing the rate of LBW by at least one-third between 2000 and 2010 as part of the Declaration and Plan of Action, ‘A World Fit for Children’ [111]. In 2004, a UNICEF and WHO report on LBW estimated that more than 20 million LBW infants were born worldwide in 2000 (15.5% of all births) [112], providing a baseline for monitoring. Global estimates published in 2019 indicate that in 2015, 14.6% (an estimated 20.5 million; 91% of those in low- and middle-income countries) of live births were LBW, compared with 17.5% (22.9 million) in 2000 [113].

In 2012, as part of WHO’s ‘Comprehensive implementation plan on maternal, infant and young child nutrition’, a new target was set to reduce LBW by 30% by 2025, compared with baseline country-level data for 2006–2010 [114]. An article in *The Lancet* Every Newborn Series (2014) described the challenges of measuring and achieving this goal, and pointed out that data gaps for morbidity and coverage and quality of care hindered efforts to plan programmes and track progress [80].

Also in 2012, *Born too soon: the global action report on pre-term birth* was published, presenting the first ever national, regional and global estimates of pre-term birth. Globally, in 2010, the pre-term birth rate was 11.1% of live births; 15 million babies were born pre-term (before 37 weeks’ gestation) and 1 million died from related complications. The pre-term birth rate in the poorest countries (12–13%) was substantially higher than high-income countries (7–9%) [9]. The latest estimates for the year 2014 (14.8 million pre-term births) now put the global pre-term birth rate at 10.6% (ranging from 13.4% in North Africa to 8.7% in Europe) [115], indicating no significant improvement since 2010.

## The way forward: aligning monitoring approaches and closing data gaps for improved maternal and newborn survival, health and well-being

The often-quoted phrase ‘What gets measured gets done’ encapsulates the relationship between measurement and action, which also underlines the reason for the focus of this review on the measurement and monitoring of maternal and neonatal mortality and morbidity and stillbirths over the past few decades – the ultimate focus is firmly on improving survival, health and well-being.

Despite major global efforts to develop MNCH targets and indicators and improve data collection and estimation methods since the 1980s (see Figure 1), and especially around the start of the MDG era, and again just before the start of the SDGs, there are still major data gaps, particularly in countries with the highest burden of mortality and morbidity and with poor routine health management information systems and limited CRVS systems. Global, regional and national estimates are now available for MMR, NMR and SBR, with modelling methods employed to enable international comparison and to provide estimates, including for countries with little or no data. But further improvements to the data sets, disaggregated analyses of these data, and refinements of the modelling methods are all vital for continued close monitoring of progress, and improved programming. The crucial missing pieces at this stage lie in the areas of maternal and neonatal morbidity; without good, widely used indicators for these, we will continue to lack an accurate picture of the burden of disease.

In September 2015, the SDGs were launched, including SDG 3 – to ensure healthy lives and promote well-being for all at all ages [116]. The 2016 *Indicator and monitoring framework for the Global Strategy for Women’s, Children’s and Adolescents’ Health*, which is aligned with and builds upon the SDG 3 targets and time frame, set MMR, U5MR, NMR, SBR and adolescent mortality rate as its five key indicators for the Global Strategy’s ‘survive’ objective, but none of its ‘thrive’ indicators have direct relevance to maternal or newborn morbidity [15].

Countdown to 2030 was launched to accelerate momentum to achieve the SDG targets for improved MNCH and to support the renewed Global Strategy [117]. Some of the suggestions for improvement in the SDG era, acknowledged in the 2015 Countdown report, include the following: establish a better set of baseline data than was available for the MDGs; improve data collection, measurement and estimation methods, especially for maternal mortality (and add data on stillbirths); use common (international) standards of measurement and reporting; and select indicators carefully for validity and reliability [63].

The SDG target of a global MMR below 70 maternal deaths per 100,000 live births will require countries to reduce their MMRs by at least an average of 6.1% each year between 2016 and 2030 [1], a much greater rate than the 2.3% average annual rate of reduction achieved during the MDG era [20]. This is an enormous challenge, especially when accurate measurement remains problematic and many deaths still go uncounted. To save newborn lives and prevent stillbirths in the coming years, Darmstadt *et al*. have called for greater focus ‘on improving coverage, quality and equity of care at birth – particularly obstetric care during labour and childbirth, and care for small and sick newborns, which gives a triple return on investment’ in terms of maternal and newborn lives and stillbirths. The authors also emphasise that progress will depend on political prioritisation for newborn lives (and for ENAP implementation), which becomes more likely when we can disseminate ‘credible data on levels of burden and intervention uptake’ [88].

## Conclusions

This review has featured the long story of the progress in monitoring improving MNH outcomes, but has also underlined current gaps and significant inequities. The many global initiatives described in this paper have highlighted the magnitude of the problems and have built the political momentum over the years for effectively addressing maternal and newborn health and well-being, with particular focus on improved measurement and monitoring. Although substantial reductions in mortality have been achieved, the enormous maternal and newborn health disparities that persist between low- and high-income countries, and within countries, show that in the SDG era there is still an urgent need to renew the focus on reducing inequities and dedicate more resources to these efforts. We must scale up evidence-based and human rights-based initiatives that work to improve the safety of pregnancy, childbirth, and the neonatal and post-partum periods.

The relevant SDG targets focus mainly on mortality reduction, but other measures are needed to capture related issues such as coverage and quality of care. And for all the MNH outcomes discussed, we still depend on estimates, as reliable data are still not collected or made available on a regular or frequent basis in many countries. In addition, the changing epidemiology of maternal mortality (with implications also for neonatal mortality and stillbirths, and maternal and neonatal morbidity) has been described by Souza *et al*. as the obstetric transition [118]. With increasing coverage of health services such as skilled birth attendants and institutional delivery, and associated changes in the epidemiology of the outcomes, there is a need to modify measurement and monitoring to be aligned with new priorities.

While this was not the focus of this review, clearly further work is needed to prioritise and harmonise measures of coverage and quality of care. MoNITOR is working to address the measurement and monitoring issues, as are other partners such as the Lancet Global Health Commission on High Quality Health Systems in the SDG Era (HQSS Commission) [119] and WHO’s Quality, Equity, Dignity Network [120]. Equally, with the Global Strategy focus on ‘survive, thrive and transform’, there is an urgent need to identify and test measures of morbidity for women and newborns. Ongoing work by WHO and others is a good start, but it needs to be prioritised – especially the development of indicators that can be routinely measured within existing systems.

Finally, monitoring progress requires strong information systems to assess different types of indicators. Currently, we rely primarily on population-based surveys conducted every two to five years (Appendix 1) and special studies to measure mortality and cause of death especially in low- and middle-income countries (Appendix 2). These surveys and studies are essential, but there is also a need for countries and donor agencies to invest in improving routine health management information systems and CRVS systems, including improvements to the availability, quality (accuracy), timeliness and the analysis and use of these data. These recommendations will ultimately lead to monitoring and measurement of what matters to improve maternal and neonatal survival, health and well-being.

## Appendix 1 Population-based household surveys gathering data to measure and monitormaternal, newborn and child health

These surveys have contributed data to national and global efforts to monitor the health and well-being of women and children, including a proportion of them which have reported on maternal and neonatal mortality.

### World Fertility Surveys (WFS)

When: Surveys conducted 1974–1983; programme ended in 1987

What: Nationally representative surveys of reproductive health and household characteristics, most including both a household and individual (female) component, often including additional topics such as water, sanitation, mortality, or economic status

Where: 42 surveys in 41 low-income countries, and 20 in high-income countries (adapted version)

Established: International Statistical Institute (ISI), the Netherlands

Funded: United States Agency for International Development (USAID), United Nations Population Fund (UNFPA) and UK Overseas Development Administration

Implemented: National agencies, often the statistical office

Comment on data use: Scientific reports published by the WFS included data on infant, child and adult mortality.

Sources: The DHS Program (https://wfs.dhsprogram.com/); Global Health Data Exchange (http://ghdx.healthdata.org/series/world-fertility-survey-wfs).

### Demographic and Health Surveys (DHS)

When: Surveys conducted 1984–present

What: Nationally representative surveys of population, health (including vaccination, treatment and prevalence of illnesses), nutrition, mortality and demographic characteristics, aiming to be repeated every five years in each country, with smaller surveys in between; the maternal mortality module was added in 1988, the year after the launch of the Safe Motherhood Initiative, and has since been implemented in 60 countries

Where: More than 300 surveys in over 90 countries

Developed and funded: USAID

Implemented: ICF International and partners (previously by Macro International, until 2009)

Comment on data use: Child, infant and neonatal mortality are routinely reported. Although data on maternal mortality are not collected in every survey, DHS have always been the main data source for estimation of maternal mortality in developing countries. The data are gathered using the direct sisterhood method (see Appendix 2), and then, maternal mortality ratios (MMRs) are calculated. These data (along with data from RHS for some countries, see below) have been used by the UN Maternal Mortality Estimation Inter-agency Group (MMEIG) to track trends in MMRs since 1990 (see Tables 2 and 3). However, underestimation of MMR using DHS data remains a challenge.

Sources: The DHS Program (https://dhsprogram.com/); Ahmed *et al*. ICF International, 2014 [121].

### Reproductive Health Surveys (RHS)

When: Surveys conducted 1992–present

What: Surveys on topics including fertility, family planning, infant and child mortality, maternal and child health (including pregnancy, delivery and post-partum care), birthweight, immunisation, breastfeeding, HIV/AIDS, adolescent and young adult sexuality, and general health practices; selected surveys have also included information on abortion, STIs, anthropometric measures, anaemia, violence against women, maternal mortality, maternal morbidity

Where: A few dozen RHS have been conducted, in countries in Latin America and the Caribbean, Africa, and eastern Europe/western Asia

Established: The U.S. Centers for Disease Control and Prevention (CDC) Division of Reproductive Health, through an agreement with USAID, established the Contraceptive Prevalence Survey (CPS) in 1975, which became the Maternal and Child Health/Family Planning (MCH/FP) Survey in the early 1980s before including additional reproductive health issues to the survey, which was then renamed as the RHS in the late 1980s

Funded: USAID

Implemented: Ministries of health and other partners, with technical assistance from CDC’s Division of Reproductive Health; the questionnaires are adapted for each country’s data requirements

Comment on data use: While the questions are similar to those in the DHS, the data have not been recoded into a standardised format. Data from RHS have been used along with DHS data for global maternal mortality estimation.

Sources: Global Health Data Exchange (http://ghdx.healthdata.org/series/reproductive-health-survey-rhs); Ahmed *et al*.; ICF International, 2014 [121].

### Multiple Indicator Cluster Surveys (MICS)

When: Surveys conducted 1995–present; since 2009 countries are supported to conduct surveys every 3 years instead of every 5 years

What: Nationally representative household surveys, conducted in rounds (currently on round 6, which was launched in 2016, to provide baseline data for the SDGs); MICS generate data with an emphasis on maternal and child health indicators, which include infant and under-five mortality rates, MMR and per cent of deliveries with a skilled attendant

Where: Over 300 MICS have been carried out in more than 100 countries

Established: UNICEF established MICS in response to the World Summit on Children (1990) and the global goals agreed at that time

Funded: UNICEF and partners provide financial assistance

Implemented by Implementing agencies (usually government agencies) customise the selected modules of the standard MICS questionnaires (designed by UNICEF) to the national setting, and implement the surveys (technical support is provided throughout by UNICEF and its MICS experts)

Comment on data use: MICS was a source of data on MDG indicators and will continue to be a source of data for SDGs. When maternal mortality data are collected, the direct sisterhood (sibling history) method is used, as for the DHS.

Source: UNICEF, 2018 (http://mics.unicef.org/); UNICEF, 2014 (https://www.unicef.org/statistics/index_24302.html).

#### General note regarding survey data on maternal mortality

As noted in the 2015 WHO report on maternal mortality trends, ‘Specialised studies indicate that there is some underreporting of maternal or pregnancy-related deaths in PMs [the proportion of deaths among women of reproductive age that are due to maternal causes] derived from sources such as population-based surveys, censuses and surveillance systems, particularly since respondents may be unaware of the pregnancy status of their sisters or other women in the household’. The estimates often have to be adjusted upwards for underreporting and/or downwards to exclude incidental/accidental deaths, and there may also be sampling errors, and errors occurring during data collection, management and analysis (WHO, 2015 and 2019) [20,1].

## Appendix 2 Challenges and advances in the measurement of maternal mortality

Due to a range of complexities, maternal mortality has been extremely challenging to measure accurately. Until the late 1980s, estimates of national and regional maternal mortality levels for low-income countries were extrapolated from relatively small studies, mostly using hospital data (i.e. not representative of the general population), while for high-income countries with civil registration systems in place, more accurate data were available earlier. With the setting of global goals (MDG Target 5.A and now SGD Target 3.1, see Table 1), all countries have needed to measure maternal mortality so that national and regional levels of MMR can be estimated and monitored to assess progress (see Appendix 1), and sub-national estimates are also needed as a basis for more effective programming and policies. The measurement methods and quality of data sources for the estimation of maternal mortality have evolved somewhat over time, but there remain many challenges in most low- and middle-income countries.

### Civil registration and vital statistics (CRVS)

In general, CRVS systems are the preferred data source and considered to be the gold standard for mortality data [1]. Ideally, all maternal and newborn deaths and stillbirths (along with all other deaths as well as births, adoptions, marriages, divorces, etc.) in a country should be recorded in the national CRVS system. The record of each death should include the age and sex of the deceased, as well as the cause of death, based on a medical certificate completed by a physician. Documentation of cause of death requires close collaboration between the CRVS system and the health sector. However, there is wide variation among countries and sometimes within countries (e.g. between provinces or states with administrative autonomy) in how CRVS systems are structured and how well they function. Some countries lack a CRVS system that is nationally representative, or any CRVS system at all, while others fail to enforce the reporting of all deaths (resulting in incomplete CRVS) and/or have high levels of misclassification of cause of death, and these reporting errors are especially likely to affect maternal and neonatal deaths, foetal deaths and stillbirths.

### Specialised and community-based studies

In the absence of a well functioning national CRVS system, many countries conduct specialised studies on maternal mortality, which are a helpful contribution to the estimation of national MMR. The task in conducting such studies is to compile accurate data on maternal deaths in a specified time period from a sample of living respondents (i.e. relatives of each deceased woman) that is large and representative enough to support a statistically reliable estimate of the frequency of this relatively rare (e.g. compared to infant or child deaths) and often sensitive or stigmatised event. Population-based household surveys require unfeasibly large sample sizes if they are to obtain statistically reliable estimates of maternal deaths by directly interviewing respondents (direct estimation), and therefore they are not cost-effective. The use of estimates with wide confidence intervals is relatively meaningless and can lead to misinterpretations of differences between locations or progress between two time points [122]. Therefore, the methods for collecting data and measuring maternal deaths using community-based studies have evolved over time and are still being refined.

The first published community-based studies on maternal mortality in low-income countries were published in 1986 and 1988. One, by Kwast *et al*. (1986), reported on a probability survey using a two-stage stratified sample to obtain a geographically representative sample in Addis Ababa, Ethiopia (1981–83), reporting an MMR of 566 per 100,000 live births for 1982–83, based on visits to 32,215 selected houses [123]. The other, by Fortney *et al*. (1988), was the Reproductive Age Mortality Survey (RAMOS) conducted in Menoufia, Egypt (1981–83) and Bali, Indonesia (1989–82), reporting MMRs of 190 and 718, respectively, among other measures [124]. The RAMOS method involves first compiling a list of deaths to women of reproductive age, either from a CVRS system (as in Menoufia) or from local leaders and community health workers (as in Bali), and then interviewing surviving family members (and supplementing this with information from physicians/hospitals if possible) and finally having this information reviewed by physicians to establish the cause of death. Adjustments are required in the later calculations since the lists of deaths will not be complete. The methods used for these and other studies, however, have serious limitations, and in general retrospective studies lead to underestimation, and prospective studies – for a relatively rare event like maternal death – are too large and costly [125].

### The sisterhood method

In 1989, Wendy Graham and colleagues introduced **the sisterhood method**, ‘a new, indirect technique for deriving population-based estimates of maternal mortality’ [126], an innovation which has been recognised as a key milestone on the road to more accurate measurement of this challenging indicator. The sisterhood method was designed to better measure maternal mortality without requiring such large sample sizes and thus it reduces costs, but it is only appropriate for use in settings with high TFR (3 +) and high maternal mortality. The major disadvantage of the indirect sisterhood method is that the results reflect a time point 10–12 years in the past [122]. With this method, all adults (age 15 +) in selected areas are asked to report how many of their ever-married sisters (born to the same mother) were alive and how many were dead, and of the latter how many had ‘died while they were pregnant, or during childbirth, or during the six weeks after the end of pregnancy’; the resulting data are used to derive the lifetime risk of maternal death, which can be translated to an MMR. These questions can be included in a multi-purpose household survey or census [126]. The sisterhood method has since been revised and refined, as indeed was anticipated in the original publication. A ‘direct sisterhood method’ variant yields estimates for a more recent time point 3–4 years before the survey, but requires the use of more questions and also a larger sample size. The DHS and MICS both use the direct variant (for more information on the DHS and MICS, see Appendix 1). As written in 1997 about the sisterhood method, ‘Both methods provide estimates of maternal mortality that should be seen as giving orders of magnitude rather than precise ratios since both can have wide margins of error (wide confidence intervals). Neither method provides a current estimate for the year of the survey. For these reasons, sisterhood studies cannot be used to monitor changes in maternal mortality nor to assess the impact of safe motherhood programmes in the short term’ [122]. According to Stanton *et al*. (2000), ‘The sampling errors associated with maternal mortality ratios are substantially larger than those associated with other frequently used DHS indicators. This lack of precision precludes the use of these data for trend analysis and has led to the recommendation that this DHS module not be used more than once every ten years in the same country’ [127]. Nevertheless, DHS data, collected using the direct sisterhood method, are used as a basis for estimation and monitoring of global MMR (using modelling techniques) by the UN MMEIG and the Institute for Health Metrics and Evaluation(IHME), for lack of any better data source (i.e. a good CRVS system) in many countries [128]. The Initiative for Maternal Mortality Programme Assessment (IMMPACT), established in 2002 under the direction of Wendy Graham, worked on (among other things) the development of new ways of measuring maternal mortality, and related tools and techniques, and also developed the Maternal Mortality Measurement Resource (MMM-R) – a web-based collection of information, materials and tools (www.maternal-mortality-measurement.org.uk/) [129].

### Maternal death surveillance and response (MDSR)

MDSR offers a method for obtaining more complete information on maternal deaths in ‘real time’ and could support better MMR estimation and stimulate more timely response and action to prevent future deaths [130]. In 2013, WHO and partners issued technical guidance on MDSR [131] ‘a continuous action cycle’ that builds on the maternal death review (MDR) approach. MDR, both community- and facility-based, was described by WHO in 2004 in *Beyond the numbers: reviewing maternal deaths and complications to make pregnancy safer* [132]. An effective MDSR system requires that maternal deaths be made a notifiable event, among others (e.g. cases of notifiable diseases). Notifications (of maternal deaths in healthcare facilities and communities) would be followed by a review to assess contributing factors and avoidability – the results of these district-level reviews feed into national-level analysis, leading to recommendations for further action, and finally response (implementation of recommendations) [131]. In countries lacking national CRVS systems, MDSR can serve as ‘a building block for a comprehensive, national-level data collection system’[1]. Since the publication of the technical guidance, MDSR has become more widely established globally and it is integral to the WHO Quality of Care initiative, the Global Strategy on Women’s, Children’s and Adolescents’ Health, the Ending Preventable Maternal Mortality (EPMM) strategy and the Every Newborn Action Plan (ENAP); its uptake and implementation are being studied, with surveys every two years and there is optimism it will contribute to eliminating preventable maternal mortality [133].WHO convenes a global technical working group (TWG) on MDSR, which was established in 2015, involving key global partners including UNFPA and UNICEF. In November 2017, this global TWG was re-launched to include perinatal audit, hence MDSR changed to MPDSR (maternal and perinatal death surveillance and response), with the aim to catalyse progress and improve MPDSR implementation globally.

### Confidential enquiry into maternal death (CEMD)

CEMD was also described in WHO’s 2004 publication, *Beyond the numbers*, defined as ‘a systematic multi-disciplinary anonymous investigation of all or a representative sample of maternal deaths occurring at an area, regional (state) or national level which identifies the numbers, causes and avoidable or remediable factors associated with them’[131]. CEMD can be used to facilitate investigation and correction of both incompleteness and misclassification in the CRVS system, with regard to maternal deaths [1]. It involves detailed expert review of all potential maternal mortality cases among women of reproductive age, to assess the accuracy of classification and to examine the circumstances of the death, thus identifying areas for action to prevent future deaths. CEMD was developed in England and Wales, where it is still used, and studies of CEMD have been conducted in a number of other countries, such as Australia, France, Ireland, Mexico and New Zealand [1].
